# Improving the Mechanical Properties of Mg-5Al-2Ca-1Mn-0.5Zn Alloy through Rotary Swaging

**DOI:** 10.3390/ma16124489

**Published:** 2023-06-20

**Authors:** Bin Li, Hao Chen, Xiangnan Ke, Guobing Wei, Qingshan Yang

**Affiliations:** 1 International Joint Laboratory for Light Alloys (MOE), Chongqing University, Chongqing 400044, China; 2 National Key Laboratory of Advanced Casting Technologies, Chongqing University, Chongqing 400044, China; 3 School of Metallurgy and Material Engineering, Chongqing University of Science and Technology, Chongqing 401331, China

**Keywords:** RE-free Mg alloy, rotary swaging, fine grains, mechanical property, microstructure

## Abstract

To meet the demand for more extensive applications of Mg alloys, a Mg-5Al-2Ca-1Mn-0.5Zn alloy without RE was prepared in this paper, and its mechanical properties were further improved by conventional hot extrusion and subsequent rotary swaging. The results show that the hardness of the alloy decreases along the radial central region after rotary swaging. The strength and hardness of the central area are lower, but the ductility is higher. The yield strength and ultimate tensile strength of the alloy in the peripheral area after rotary swaging reach 352 MPa and 386 MPa, respectively, while the elongation remains at 9.6%, exhibiting better strength–ductility synergy. The grain refinement and dislocation increase caused by rotary swaging promoted strength improvement. The activation of non-basal slips during rotary swaging is an important reason for the alloy to maintain good plasticity while improving strength.

## 1. Introduction

With the rise in global temperature, the Earth on which human beings live is being irreversibly damaged, and the living environment is under great threat [1,2]. Therefore, under the requirement of “double carbon”, developing new materials and applying new technologies and processes are of great significance for achieving green development [3,4]. As the lightest structural metal material, magnesium (Mg) alloys have many advantages such as high specific strength, high heat dissipation, recyclability, etc. [5,6,7]. Magnesium is widely used in aerospace, transportation, electronics, medical and other fields, and is known as “the most developable green material in the 21st century” [8]. Therefore, it is an effective way to reduce carbon emissions to reasonably apply Mg alloys to various fields [4].

Compared with steel, aluminum, titanium, and other structural materials, the strength and ductility of Mg alloys are relatively low [4,9,10]. Alloying and grain refinement are the main ways to improve the mechanical properties of Mg alloys [11]. Previous studies have shown that the addition of rare earth (RE) elements can significantly enhance the mechanical properties of Mg alloys [12,13]. However, in general, RE elements are not only dense but also expensive, which is not suitable for large-scale commercial applications. Therefore, RE-free Mg alloys are also one of the important ways to research Mg alloys at present [14].

Mg-Al-Zn-series alloys, like AZ31 and AZ91, are the most extensively used commercial Mg alloy in engineering [15,16,17]. The addition of Ca can not only enhance the strength of Mg alloys by refining the grain but also weaken the texture and improve their ductility [18]. Moreover, the Mn element is a microalloy which is also widely used in Mg alloys for several reasons. Firstly, Mn can eliminate Fe in Mg alloy melt, thus achieving the purpose of purification [19,20]. Moreover, the addition of Mn can reduce stacking faults energy and improve ductility [21]. In addition, the Al-Mn phase with a high melting point will not decompose during hot deformation, improving the thermal stability of Mg alloys [21,22,23]. Therefore, the Mg-5Al-2Ca-1Mn-0.5Zn alloy was designed to improve the mechanical properties of Mg alloys (denoted as AXMZ5210).

It is widely known that Mg alloys cannot achieve a high strength only through alloying [24,25]. Plastic deformation is a common means to enhance the strength of metallic materials owing to the size effect [24]. Common plastic deformation methods, such as rolling, extrusion, and forging, cannot obtain the large plastic deformation degree and ultrafine grains [26,27]. Therefore, various methods of severe plastic deformation (SPD) were invented to produce polycrystalline metals with ultrafine or nanosized grains [28,29]. Compared with traditional SPD methods such as equal channel angular pressing (ECAP), high-pressure torsion (HPT), accumulated roll bonding (ARB), etc. [30,31], rotary swaging (RS) is a cost-effective process owing to its advantages of low forming force required for processing and excellent mechanical properties of products [32,33]. As the sample passes through the RS mold, it is subjected to continuous pulse pressure along the radial direction of the mold, which can produce higher hydrostatic stress, effectively refine the grains, and obtain a work hardening effect. Yang et al. [24] increased the ultimate tensile strength (UTS) of the Mg-4Li alloy to 405 MPa using RS. Chen et al. [16] used cryogenic RS to prepare AZ31B alloy with an average grain size of only ~93 nm, which exhibits ultra-high UTS (560 MPa) and yield strength (YS, 495 MPa). Wan et al. [7] obtained equiaxed grains with an average grain size of 80 nm after four passes of RS of the Mg-Gd-Y-Zr alloy, and a UTS of up to 710 MPa after aging. However, there are still few reports about RE-free Mg-Al-Mn-series alloys prepared by RS, especially on the microstructure evolution during RS and the strengthening mechanism.

In the present study, AXMZ5210 alloy rods were prepared via conventional hot extrusion and RS. A high-strength Mg alloy with a UTS of 386 MPa and an elongation of ~10% was obtained by RS. The microstructure inhomogeneity during RS was investigated, and the effect of RS on mechanical properties was discussed.

## 2. Materials and Methods

**Figure 1:**
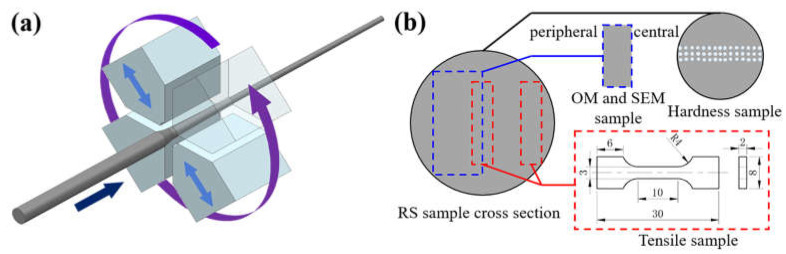
(**a**) Schematic illustration of rotary swaging process, and (**b**) the cutting parts for different purposes (unit: mm).

The AXMZ5210 alloy was prepared using commercial pure Mg, pure Zn, pure Al, Mg-3 wt.% Ca, and Mg-3 wt.% Mn master alloy. The alloys were weighed according to design composition and then were melted at 780 °C in a resistance furnace with a protection mixed gas of CO_2_ and SF_6_. After all of the alloys in the crucible were completely melted, the melt was stirred to make the alloy elements diffuse evenly. Finally, the AXMZ5210 ingots were fabricated by direct-chill casting, and then subjected to solid solution treatment at 350 °C for 11 h, immediately followed by water quenching. Then, the ingots were cut into a round rod with a size of Φ80 mm × 50 mm. After pre-heating at 360 °C for 2 h, the ingots were extruded to Φ16 mm rods at 360 °C. After extrusion, the alloy rod was subjected to 5 passes of RS at room temperature. The working principle of RS is shown in Figure 1a. The striking frequency of RS is 100 times/min, the axial movement speed of the sample is ca. 60 mm/s, and the reduction is 1 mm for the first pass and ca. 0.2 mm for each subsequent pass. Finally, an alloy rod with a diameter of ca. 14.3 mm and a strain of ca. 20.2% was obtained.

Dog-bone-shaped tensile samples with a gauge length of 10 mm were prepared along the extrusion direction (ED), as shown in Figure 1b. The microhardness of the RS cross-section was tested by a 310HVS-5 hardness tester. The load was 100 gf, the dwelling time was 10 s, and the distance between adjacent indentations was 1 mm. The phase composition of the alloy was determined by X-ray diffraction (XRD, D/max-2500pc, Cu Kα radiation) at the scan rate of 3° min^−1^ approximately from 10 to 90°. The tensile tests of the alloys were conducted on a SANS CMT-5105 tensile tester with a strain rate of 1 mm min^−1^. The microstructure was characterized by optical microscopy (OM, OLYMPUS PMG3, Tokyo, Japan) and scanning electron microscope (SEM, JEOL JSM-7800F, Tokyo, Japan) equipped with an Oxford Aztec electron backscatter diffraction (EBSD, Abingdon, UK) detector and an energy-dispersive spectrometer (EDS). The grain size distribution histogram of OM images was obtained by using IPwin32 software (Image-Pro Plus 6.0). EBSD data were analyzed in detail using HKL Channel 5 software (Aztec 3.1).

## 3. Results and Discussion

### 3.1. Microstructure Analyses

**Figure 2:**
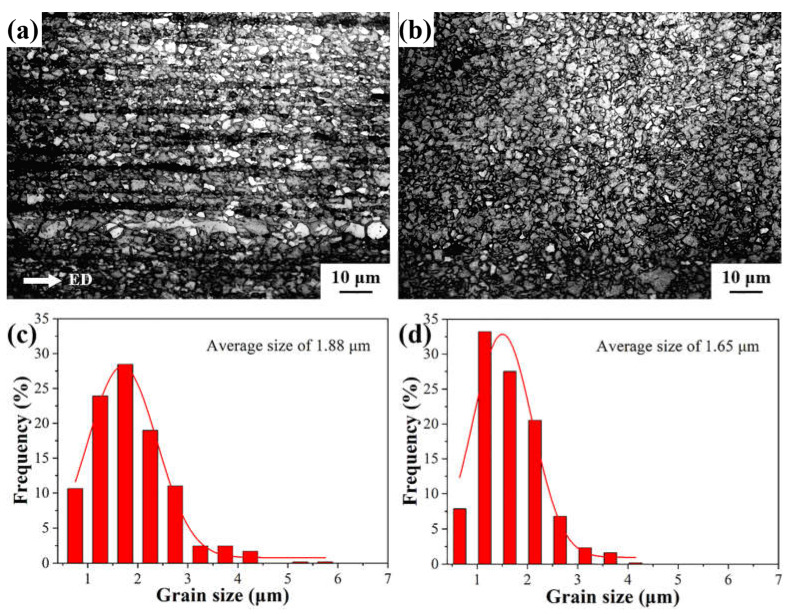
The OM images and grain size distribution of AXMZ5210 alloy in (**a**,**c**) the central area and (**b**,**d**) the peripheral area of RS.

The OM images of the as-swaged AXMZ5210 alloy are shown in Figure 2a,b. The original grain size after extrusion is ca. 2.42 μm, and more details can be found in our previous study [34]. Compared with the as-extruded AXMZ5210 alloy [34], the grain size of the alloy is further refined after RS (Figure 2c,d). The average grain size in the central and peripheral areas of RS is reduced by 22.3% and 31.8%, respectively, from the original extruded alloy. Nevertheless, the microstructure of the swaged alloy is not homogeneous. The microstructure of the central area has a typical bimodal structure, which is composed of fine equiaxed grains and large grains elongated along the ED. The grain size of the peripheral area is finer (1.65 μm) because it is first subjected to continuous pulse pressure along the radial direction of the mold and has higher strain [33,35]. Its organization is also more homogeneous than the central area.

**Figure 3:**
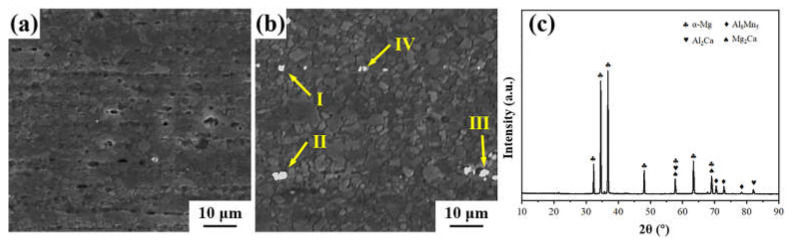
The SEM images of AXMZ5210 alloy in (**a**) the central area and (**b**) the peripheral area of RS, and (**c**) XRD pattern of AXMZ5210 alloy.

Our previous study [34] showed that the as-extruded AXMZ5210 alloy has more second-phase particles. However, the number of white second-phase particles in the SEM images of the as-swaged AXMZ5210 alloy shown in Figure 3a,b are extremely small. During the RS process, the second phase particles may be crushed and partially dissolved into the Mg matrix due to the interaction of high temperature and stress [22]. The EDS results in Figure 3b are shown in Table 1, and the positions in the table correspond to Figure 3b. Combined with the XRD results shown in Figure 3c, it can be determined that these white second-phase particles are mainly composed of Al_8_Mn_5_, with a relatively small amount of (Mg, Al)_2_Ca [22,34].

**Figure 4:**
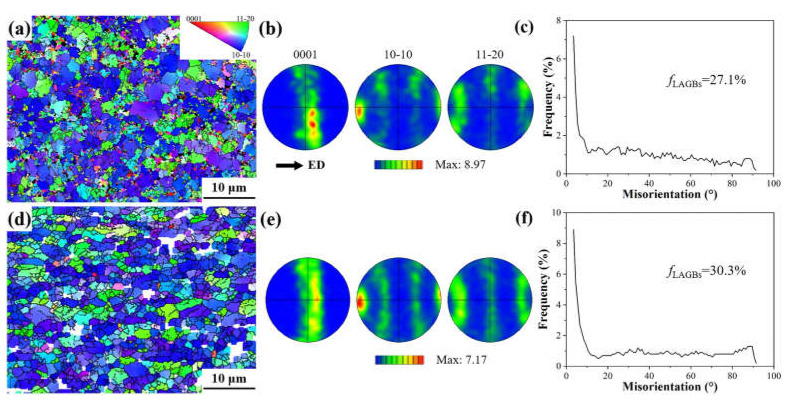
IPF maps, misorientation angle distributions, and the pole figures with maximum intensity of AXMZ5210 alloy. (**a**–**c**) The central area and (**d**–**f**) the peripheral area of RS.

To further analyze the microstructure inhomogeneity of the as-swaged AXMZ5210 alloy, EBSD tests were performed on the alloys in the central and peripheral areas. As shown in Figure 4a,d, the microstructure characteristics obtained by EBSD are consistent with the OM results. In addition, it is seen that after RS, most of the grains are biased towards the {10$\bar{1}$0} plane, with an obvious preferred orientation. The pole figures of Figure 4b,e show that the as-swaged AXMZ5210 alloy has a typical basal fiber texture with the <10$\bar{1}$0> direction parallel to ED. Figure 4c,f show the misorientation angle distribution of the swaged alloy. It can be seen that the proportion of low-angle grain boundaries (LAGBs, 2° ≤ θ ≤ 15°) in the peripheral area (30.3%) is significantly higher than that in the central area (27.1%). The proportion of LAGBs in the as-extruded AXMZ5210 alloy is only 11.4% [34]. Generally, the low-angle misorientations are mainly the result of dislocation activity, which means that the higher the proportion of LAGBs, the higher the dislocation density [17,27]. The RS usually causes dislocation multiplication, and the peripheral area has greater plastic deformation, which ultimately results in a higher ratio of LAGBs [22].

**Figure 5:**
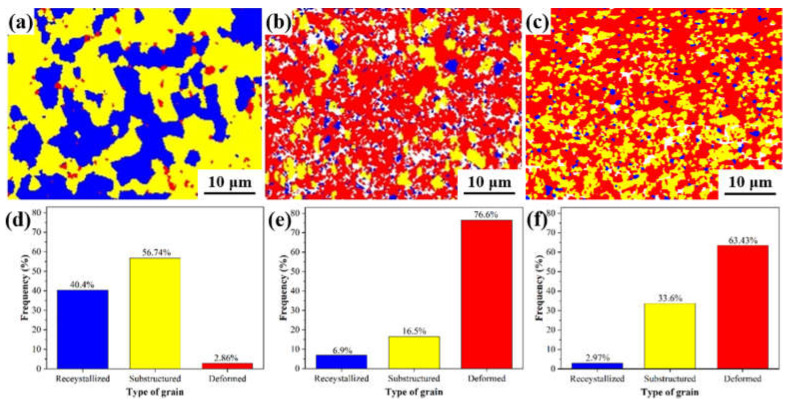
EBSD maps and grain type distribution of recrystallized grains (blue), sub-structured grains (yellow), and deformed grains (red) of AXMZ5210 alloy. (**a**,**d**) as-extruded, (**b**,**e**) the central area, and (**c**,**f**) the peripheral area of RS.

Figure 5 quantitatively analyzes the area fraction of recrystallized grains, deformed grains, and sub-structured grains in the original extruded alloy and the center and peripheral areas of the swaged alloy. The as-extruded alloy has a higher recrystallized material because its higher processing temperature makes recrystallization easier [36,37]. Due to the RS being performed at room temperature, the deformed grains occupy the main area in the as-swaged alloy, while the degree of recrystallization is rather low. On the other hand, in the as-extruded alloy, the sub-structured grains occupy the main area. After RS, the sub-structured grains in the alloy decrease significantly, especially in the central area of RS. The formation of sub-structures is related to dislocation interaction [38]. Our previous study [34] showed that in the as-extruded AXMZ5210 alloy, a large number of dislocations accumulated inside the grains due to the presence of nanoscale Al-Mn phase in the matrix, which may be related to the existence of more sub-structured grains in the extruded alloy [39,40]. In the subsequent RS, the further accumulation of dislocations due to the higher strain may force the sub-structure to transform to high-angle grain boundaries (HAGBs), thus refining the grain size [17,40].

### 3.2. Mechanical Properties

**Figure 6:**
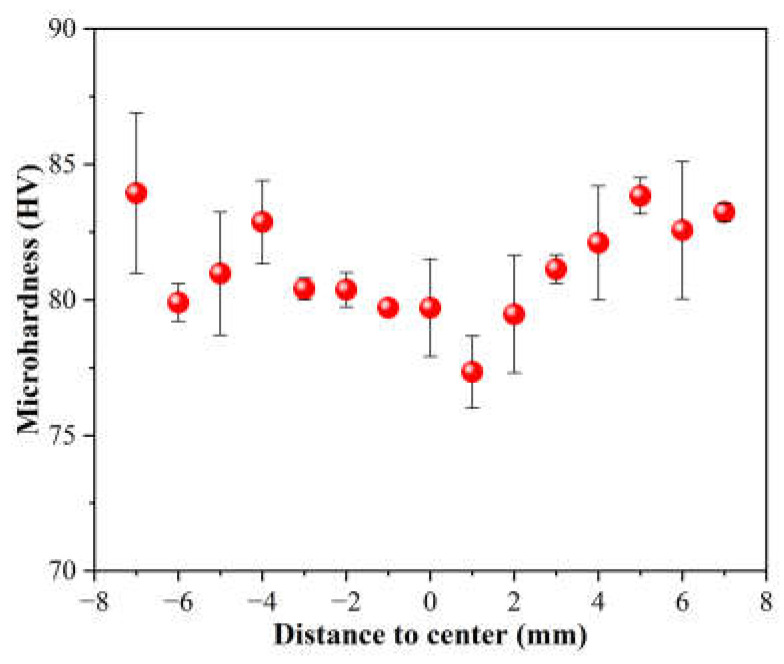
Cross-sectional microhardness distribution after RS.

The microhardness distribution of the cross-section of the as-swaged AXMZ5210 alloy is shown in Figure 6. It can be seen that the microhardness of the peripheral area is generally higher than that of the central area, showing an approximate V-shaped distribution. The highest microhardness appears in the peripheral area at a distance of ca. 7 mm from the center area, which is 84 HV, and the lowest in the center area is 77 HV. The reason for the higher microhardness in the peripheral area will be discussed later.

**Figure 7:**
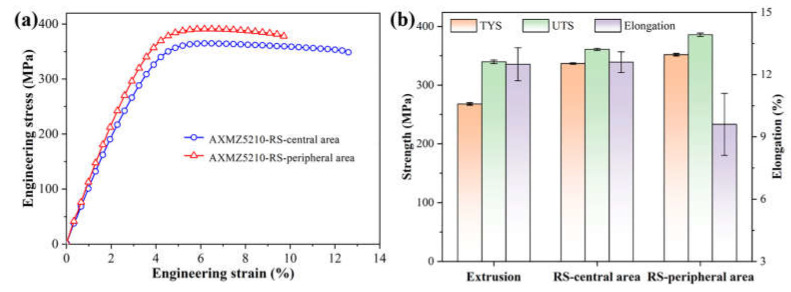
(**a**) The tensile engineering stress–strain curves and (**b**) comparison of mechanical properties of AXMZ5210 alloy [34].

The stress–strain curves for the as-swaged AXMZ5210 alloy at room temperature and a comparison of the mechanical properties with the original as-extruded alloy are shown in Figure 7. Compared with the central area of RS, the peripheral area has higher YS (352 MPa) and UTS (386 MPa), while still having a 9.6% elongation. The strengthening effect of the central area is weak, and its YS (337 MPa) and UTS (361 MPa) are 15 MPa and 25 MPa lower than the peripheral area, respectively, but it has higher elongation (12.6%). Our previous study [34] showed that the as-extruded AXMZ5210 alloy has better comprehensive mechanical properties compared to many as-extruded RE-free alloys (e.g., AZ31, Mg-Zn-Ca alloy). After RS, its YS and UTS were further improved, but the elongation did not decrease significantly (Figure 7b). This indicates that the RE-free AXMZ5210 alloy prepared by RS also has good strength–ductility synergy.

**Figure 8:**
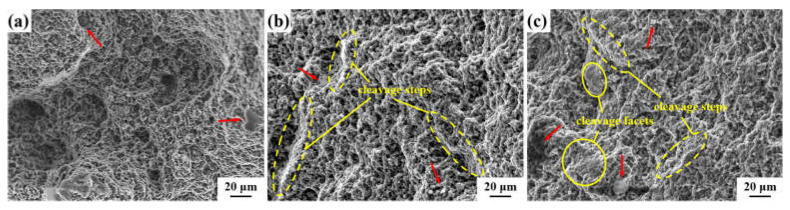
Fractographs of the AXMZ5210 alloy. (**a**) As-extruded, (**b**) the central area, and (**c**) the peripheral area of RS (red arrows indicate fine particles).

The tensile fracture surfaces of the AXMZ5210 alloy in different states at room temperature are shown in Figure 8. Compared with the original as-extruded alloy, there are still small dimples in the tensile fracture after RS, but the depth becomes shallower, which reflects that the ductility is reduced, consistent with the mechanical properties of Figure 7b. Meanwhile, the tensile fracture morphology of the as-swaged alloy shows a cleavage step as a whole, as shown in the yellow wireframe region in Figure 8b,c, which is between ductile fracture and brittle fracture. On the other hand, the presence of fine particles was also found in the tensile fracture (red arrow). According to the results of XRD and SEM, these particles are most likely unbroken second phases and can act as a source of cracks to reduce the ductility of the alloy.

### 3.3. Strengthening Mechanism

In general, the excellent mechanical properties of SPD-treated Mg alloys are usually related to the ultrafine-grained microstructure generated during SPD [17,31]. According to the Hall–Petch relationship, the enhanced YS due to fine grain strengthening can be calculated using the following equation [14,41]:

(1) $$\sigma_{\mathrm{gbs}}{=\sigma }_{0}{+kd}^{-0.5}$$

where *σ*_gbs_ is YS enhanced by fine grain strengthening, *d* is the average grain size, and *σ*_0_ (the value is 54 MPa [19]) and *k* (the value is 212 MPa μm^1/2^ [23]) are constants related to the material. Taking the alloy in the peripheral area with a *d* of 1.65 μm as an example, it is calculated that the *σ*_gbs_ is about 219 MPa. It can be seen that fine grain strengthening is the main reason for the strength improvement after RS.

The high dislocation density generated in the RS process is also an important reason for the strength improvement of the as-swaged alloy [22]. High dislocation density can induce higher work hardening rate, which is beneficial to the strengthening of the alloy [10,33]. In the as-swaged AXMZ5210 alloy, the decrease in grain size makes the average distance of dislocations decrease, which in turn leads to a large number of dislocations accumulating because grain boundaries can hinder dislocation movement [42]. Moreover, it was found by SEM that the second-phase particles might be broken and dissolved into the Mg matrix during the RS process (Figure 3a,b). The second phase particles themselves can hinder the dislocation motion, and when dissolved into the matrix, it is easy to cause lattice distortion, providing additional resistance for dislocation glide promoting dislocation nucleation [42,43,44].

Mg alloys with an HCP structure usually have poor ductility, and the opening of a <c + a> pyramidal slip or the coordinated deformation of twins is particularly important for the improvement of the ductility of Mg alloys [33,45]. To coordinate the deformation during the RS process, twins are activated to adapt to uniform deformation at low strain [22,33]. With increasing strain, massive dislocation arrays are formed in the twins, which are refined into subgrains [40]. As the rotary RS process continues, these subgrain boundaries are gradually transformed into HAGBs [22,40]. As a result, the average grain size of the as-swaged alloy is significantly reduced.

**Figure 9:**
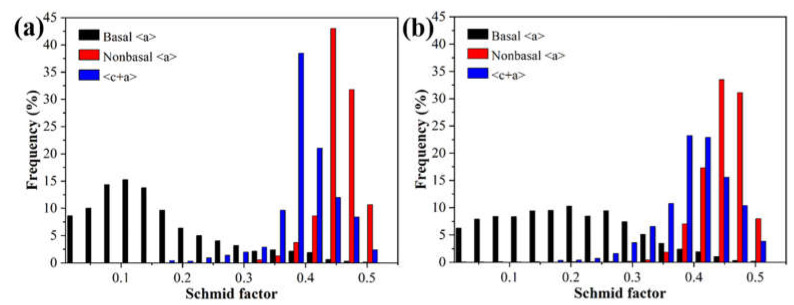
Schmid factor of the various slips in AXMZ5210 alloy. (**a**) The central area and (**b**) the peripheral area of RS.

It has been shown that <c + a> slip and twinning are sensitive to grain size [11,33]. When the grain size is less than 5μm, the activity of twins is restricted [11,29], which is beneficial in the tensile process, because compression twins and double twins can easily cause stress concentration [6,34], while tension twins cannot provide independent slip systems [13]. On the other hand, fine grain is beneficial to the activation of non-basal slip [40]. To investigate the effect of RS on the plasticity of the AXMZ5210 alloy, the Schmid factor (SF) of different slip systems was statistically analyzed by EBSD, as shown in Figure 9. It can be seen that the SF of both non-basal <a> and <c + a> slips of the as-swaged alloy are higher than that of the basal <a> slip. Compared with the as-extruded AXMZ5210 alloy [34], the SF of non-basal <a> and <c + a> slips of the as-swaged alloy are higher, which indicates that RS is beneficial to the activation of non-basal <a> and <c + a> slips [7,24]. The activation of non-basal slip can provide sufficient independent slip systems for Mg alloys during the tensile process, which helps the as-swaged AXMZ5210 alloy to maintain high ductility [13]. The SF of non-basal slip in the central area is significantly higher than that in the peripheral area, which implies better ductility, consistent with the mechanical properties in Figure 7.

In addition, the strength and hardness of the as-swaged AXMZ5210 alloy are higher in the peripheral region, and the strength and hardness of the central region are lower, but the ductility is better. This situation is usually more common in W alloys [21]. During the RS process, the peripheral area will first deform, because the alloy rod surface is in direct contact with the mold. It is well known that Mg alloys have good damping properties. Therefore, the pulse pressure generated during RS may be heavily consumed by the Mg matrix, resulting in less stress transferred to the central area and eventually greater strain in the peripheral area. This may explain the difference in microstructure between the central and peripheral areas shown in Figure 2, Figure 3, Figure 4 and Figure 5. However, the damping capacity of the AXMZ5210 alloy was not investigated in this work, and subsequent studies are needed to confirm this conjecture.

## 4. Conclusions

The mechanical properties of the RE-free Mg-5Al-2Ca-1Mn-0.5Zn alloy were improved by rotary swaging, and the microstructure evolution and strengthening mechanism during rotary swaging were discussed. Major conclusions can be drawn as follows:

(1) The grain size is obviously refined, and the proportion of low-angle grain boundaries increases significantly after rotary swaging, especially in the peripheral area. The second phase particles are mainly composed of Al_8_Mn_5_, which are broken and partially dissolved into the matrix during rotary swaging.
(2) The YS and UTS of the alloy are significantly improved after rotary swaging, which can be mainly attributed to fine grain strengthening. The strengthening effect of the peripheral area is more significant, and its YS and UTS reach 352 MPa and 386 MPa, respectively.
(3) The as-swaged Mg-5Al-2Ca-1Mn-0.5Zn alloy still maintains high plasticity (elongation of 9.6%) with good strength–ductility synergy while improving strength, which is related to the activation of non-basal slips during rotary swaging.

## Figures and Tables

**Table 1:** EDS results of the second phase particles marked by arrows in Figure 3.

Positions	Mg (at.%)	Al (at.%)	Ca (at.%)	Mn (at.%)	Zn (at.%)	Phase
Ⅰ	79.3	12.3	0.2	7.9	0.3	Al_8_Mn_5_
Ⅱ	69.9	15.4	0.2	14.3	0.2	Al_8_Mn_5_
Ⅲ	82.1	10.6	0.1	7.1	0.1	Al_8_Mn_5_
Ⅳ	87.7	5.6	1.1	5.5	0.1	Al_8_Mn_5_, (Mg, Al)_2_Ca

## Data Availability

Not applicable.
